# Using Acellular Bioactive Extracellular Matrix Scaffolds to Enhance Endogenous Cardiac Repair

**DOI:** 10.3389/fcvm.2018.00035

**Published:** 2018-04-11

**Authors:** Daniyil A. Svystonyuk, Holly E. M. Mewhort, Paul W. M. Fedak

**Affiliations:** Section of Cardiac Surgery, Department of Cardiac Sciences, Cumming School of Medicine, Libin Cardiovascular Institute of Alberta, University of Calgary, Calgary, AB, Canada

**Keywords:** extracellular matrix, biomaterials science, cardiovascular diseases, regeneration mechanisms, cardiovascular surgery

## Abstract

An inability to recover lost cardiac muscle following acute ischemic injury remains the biggest shortcoming of current therapies to prevent heart failure. As compared to standard medical and surgical treatments, tissue engineering strategies offer the promise of improved heart function by inducing regeneration of functional heart muscle. Tissue engineering approaches that use stem cells and genetic manipulation have shown promise in preclinical studies but have also been challenged by numerous critical barriers preventing effective clinical translational. We believe that surgical intervention using acellular bioactive ECM scaffolds may yield similar therapeutic benefits with minimal translational hurdles. In this review, we outline the limitations of cellular-based tissue engineering strategies and the advantages of using acellular biomaterials with bioinductive properties. We highlight key anatomic targets enriched with cellular niches that can be uniquely activated using bioactive scaffold therapy. Finally, we review the evolving cardiovascular tissue engineering landscape and provide critical insights into the potential therapeutic benefits of acellular scaffold therapy.

## Introduction

Heart failure is a growing epidemic that is predicted to disable 1 in 5 Americans in their life time (1). Despite the prevalence of heart failure, effective treatment options remain limited. Pharmacological interventions can improve symptoms and prolong survival, but are unable to promote functional recovery of cardiomyocytes lost to injury (2). Organ transplantation remains the only curative option but a disparity between donor heart supply and patient demand coupled with the need for immunosuppressive therapy makes this an ineffective solution to address the growing needs of the heart failure population (3). Durable mechanical support therapies continue to evolve and improve but complications for destination therapy patients are a concern.

As our understanding of the factors and mechanisms that regulate heart structure and function have improved, the concept of engineering cardiovascular tissues to restore heart function has rapidly advanced (4, 5). Whole organ regeneration is the ultimate goal of tissue engineering but at present exists only as a futuristic possibility. Early tissue engineering approaches using stem cell and gene therapy have shown promise, but remain fraught with translational hurdles. As such, there has been an increasing shift in focus towards utilizing tissue engineering strategies that can stimulate repair by modulating the host-substrate microenvironment and enhancing endogenous tissue repair processes (6).

In this review, we focus on the translational limitations of contemporary cardiac regenerative approaches and describe how acellular bioactive ECM scaffolds may provide an effective solution. Specifically, we outline important anatomical and cellular targets that may benefit from bioactive scaffold therapy and provide insights into the future of cardiovascular tissue engineering and its translation into viable clinical applications.

## Early Tissue Engineering Strategies Towards Cardiac Regeneration

The field of cardiovascular tissue engineering was born out of a need to design functional substitutes for tissue that was presumed irreversibly damaged. Leveraging the plasticity of stem cells and direct genetic manipulation became popular options to achieve this goal.

The ability to effectively isolate and expand endogenous stem cells offered the exciting promise of leveraging the cells’ inherent regenerative capacity to treat cardiovascular disease (7). Over the past decades there has been significant enthusiasm within the scientific community for cell-therapies based on a foundation of encouraging preclinical evidence. Why is it that cell-mediated regeneration remains absent from conventional treatment modalities? Part of the problem lies in the biology surrounding exogenous cell delivery to the microenvironment of a failing heart. Damaged myocardium lacks the necessary structural and biological microenvironment to support proper cell health and function. Accordingly, it is no surprise that stem cell survival and engraftment is poor and this remains a dominant issue preventing effective clinical translation (8). Interestingly, the benefits of cell therapy are well documented in preclinical animal models despite the fact that cells are delivered to similar hostile microenvironments in the heart. Long term donor cell engraftment and survival is poor yet functional myocardial recovery is readily observed. These findings represent a paradigm shift in our understanding of the cell-mediated therapeutic effect, indicating that the benefits of cell therapy may lie in their ability to act as source of regenerative and reparative paracrine factors (9, 10).

Gene therapy allows targeted control of specific molecular pathways, typically through adenoviral vectors, that can restore lost functionality or enhance endogenous cardiac repair processes (11). Contemporary gene therapy approaches have targeted a number of cardiovascular systems, including: cell metabolic activity, calcium regulation, vasculogenesis, and stem cell activation (12). The concept of targeting single genes to drive critical repair pathways toward functional recovery is exciting but clinical outcomes of gene therapy have been mostly unsuccessful. Of the five cardiac gene therapy clinical trials published to date, all five have shown safety but failed to meet primary efficacy endpoints (13–17). Indeed, targeting a single gene in a pathway that involves multiple complex molecular mechanisms is unlikely to yield appreciable clinical benefit. Interestingly, trials that aimed to genetically bolster stem cell recruitment to the myocardium showed benefit in a cohort of patients with advanced ischemic cardiomyopathy (16).

The lessons learned from attempts at gene therapy for heart failure are important: enhancing targeted molecular pathways and signalling mechanisms in failing myocardium can have substantial therapeutic benefits (18). This challenged the notion that tissue engineering must necessarily be an “outside-in” approach and instead, argued that tissue engineering can occur from within by rescuing and/or stimulating endogenous repair pathways.

## Leveraging Acellular Bioactive Scaffolds Towards Cardiac Regeneration

The paracrine hypothesis of cell therapy and direct genetic manipulation of endogenous repair mechanisms highlights that a failing heart can be primed toward tissue regeneration and repair by altering the signalling environment of the host cells. Acellular bioactive scaffolds serve as niche signalling microenvironments that may be used toward driving cardiac repair (19). While such scaffolds can be synthetic or semi-synthetic and injectable or non-injectable, this report will focus on extracellular matrix (ECM)-based patch biomaterials.

The mainstay of acellular bioactive materials is the extracellular matrix, a structural scaffold that has all of the necessary cues and signals to support proper cell health, function and tissue repair processes (4). Some studies have utilized a simple ECM scaffold consisting of either type I collagen or gelatin as a vessel to deliver a single protein or cell type (20, 21). Conversely, more complex ECM scaffolds may be derived through the decellularization of biological tissue. These scaffolds may exert bioactive effects by way of growth factor reservoirs, matricellular proteins and complex ultrastructural compositions (22, 23).

Early studies characterizing acellular biological tissues have shown that the decellularization process does not disrupt native bioactive constituents present in the ECM scaffolds, such as FGF-2 and VEGF (24). Additionally, degradation products produced by the remodeling of the ECM materials by the host tissue has been shown to affect endogenous cell activity (25). As such, decellularized ECM scaffolds from highly regenerative organs, like the gastrointestinal system, may be used to circumvent the limited regenerative capacity of the heart (26). Following decellularization, the bioactive properties of the ECM can be leveraged without the underlying safety concern of an adverse immunogenic response (22, 24). In fact, Dziki and colleagues demonstrated that acellular bioactive scaffolds may influence macrophage polarization away from a pro-inflammatory M1 phenotype towards a pro-reparative M2 phenotype (27). To date, there have been an abundance of convincing preclinical studies that outline the cardioprotective benefits of ECM biomaterials in the heart (28–32).

**Figure 1:**
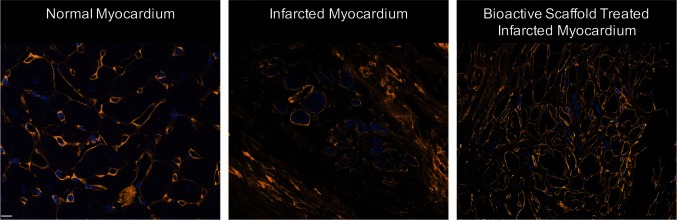
Representative images of normal myocardium, infarcted myocardium from a sham, and infarcted myocardium following surgical implantation of bioactive scaffold on the epicardial surface (blue = nucleus, orange = collagen). The infarct area of bioactive scaffold-treated animals showed less collagen density and ECM architecture more consistent with normal cardiac tissue (Reprinted from The Journal of Thoracic and Cardiovascular Surgery, Vol 147/Issue 5, Holly EM Mewhort, Jeannine D Turnbull, Christopher Meijndert, Janet MC Ngu, Paul WM Fedak, Epicardial infarct repair with basic fibroblast growth factor–enhanced CorMatrix-ECM biomaterial attenuates postischemic cardiac remodeling, 1650–1659., Copyright 2014, with permission from Elsevier) (28).

**Figure 2:**
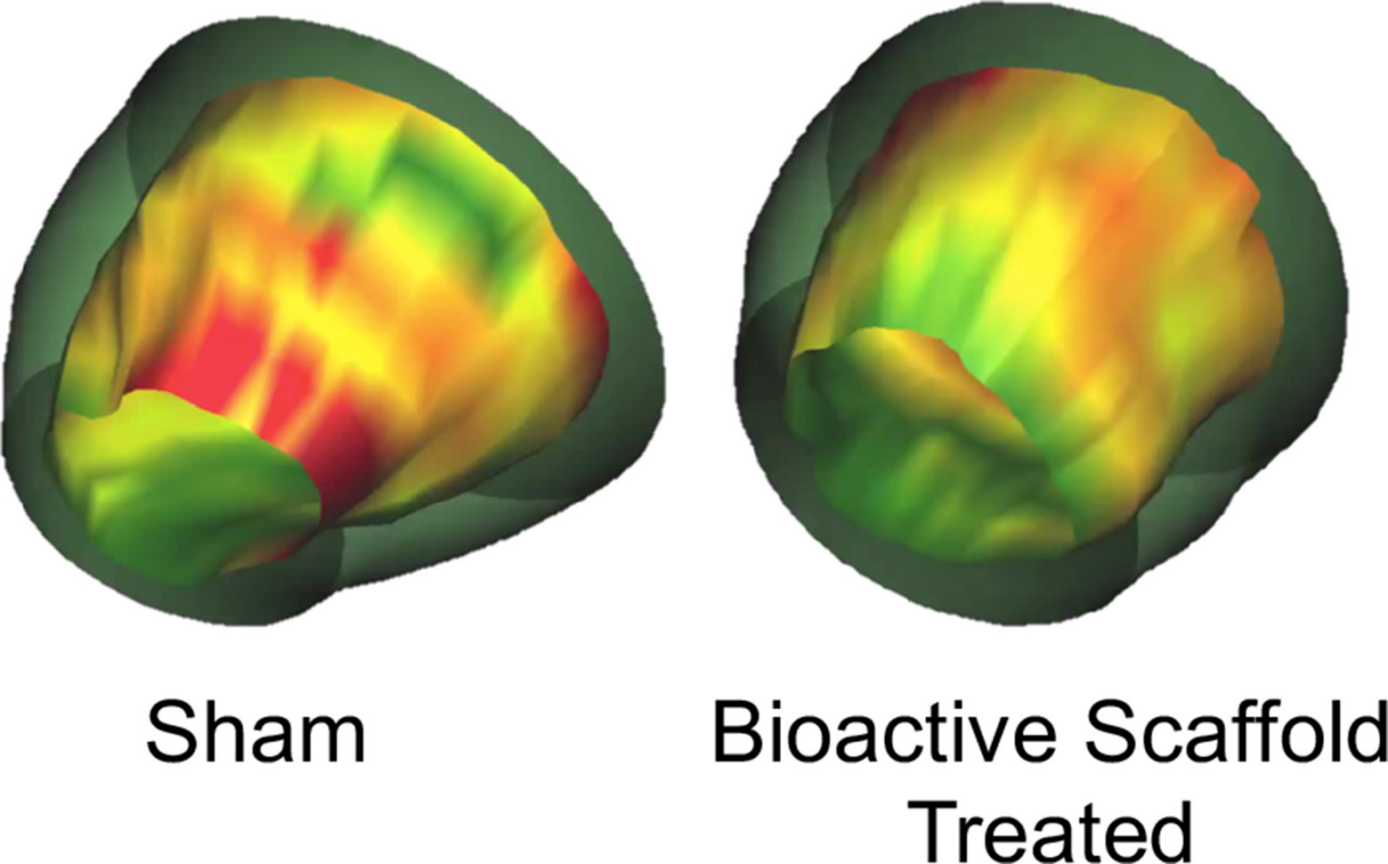
3-D images of the LV reconstructed from MRI data depicting wall thickening in sham versus bioactive scaffold-treated animals 6 weeks after the initial ischemic event (green = normal, yellow = hypokinetic, red = akinetic). Bioactive scaffold treatment resulted in regional improvement in myocardial function (29).

**Figure 3:**
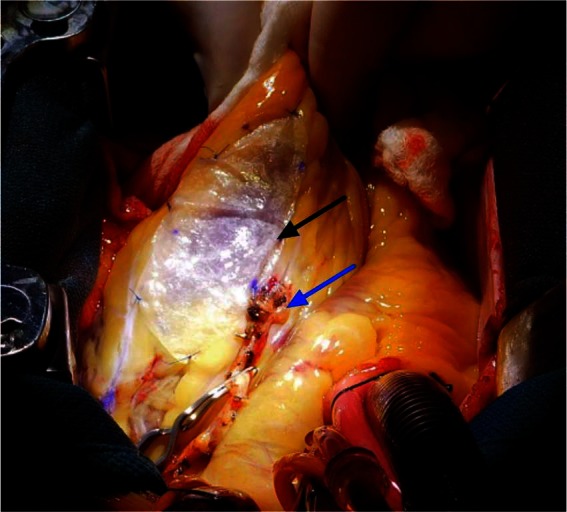
Representative image of surgical implantation of a bioactive scaffold at the time of revascularization surgery. Patients were selected for bioactive scaffold therapy in adjunct with CABG and followed by serial cardiac MRI up to six months following surgery. Black arrow indicates acellular bioactive scaffold. Blue arrow indicates bypass graft.

Our group has explored epicardially implanted acellular bioactive scaffolds across a number of clinically relevant models of ischemic injury. We first established efficacy in a rodent chronic heart failure model where we demonstrated that surgically implanted ECM scaffolds can attenuate infarct expansion and LV remodeling while simultaneously improving cardiac function (Figure 1). Importantly, we demonstrated that bioactive scaffolds can be further enhanced with exogenous growth factors highlighting its capacity as a platform therapy (28, 33). Using a large preclinical porcine model of ischemia-reperfusion, we were able to observe regional myocardial improvements by serial cardiac MRI following surgical implantation of bioactive scaffolds during the acute stage post-MI (Figure 2). Interestingly, histological examination of the infarct area in bioactive scaffold-treated animals showed small arteriole formation next to islands of surviving cardiomyocytes (29). We later confirmed that these beneficial effects are due to bioactive constituents present within the scaffolds and were not the result of a passive myocardial restraint effect (34). Collectively, these findings have given us insight into the optimal therapeutic window for bioactive scaffold therapy and suggest that the greatest benefit may be as an adjunct to surgical macroscopic revascularization where hibernating myocardium is perfused by bioactive scaffold-mediated microvascular formation. At present, we are completing a first-in-man pilot clinical feasibility trial (ClinicalTrials.gov ID: NCT02887768) where acellular bioactive scaffolds are surgically implanted at the time of CABG surgery (Figure 3).

Although passive mechanical restraint has been shown to benefit functional recovery of the failing heart, this was not theprimary mechanism observed in our studies as scaffold implants did not alter ventricular compliance and vasculogenesis was observed (35, 36). How is it then that acellular bioactive scaffolds can induce adaptive tissue remodeling and improve function? Emerging evidence has identified the plasticity and regenerative capacity of endogenous cells and anatomic structures of the heart (34, 37). Surgically implanted bioactive scaffolds introduce a new signalling microenvironment in the heart that may potentiate these innate regeneration processes. Specifically, altering the function of the epicardium and matrix-modulating cardiac fibroblasts may demonstrate how nature’s own platform can be leveraged to promote endogenous cardiac regeneration.

### The Epicardium as an Anatomic Niche for Endogenous Repair

Over the past decade, insights from vertebrate studies have identified the epicardium as the key structure responsible for their high cardiac regenerative capacity (38). Understandably, targeting the epicardium for tissue regeneration has been the subject of great therapeutic interest.

The epicardium is the outermost mesothelial layer of the heart surrounding the myocardium (39). In early development, the epicardium is a source of progenitor cells that undergo epithelial to mesenchymal (EMT) transition to yield vascular smooth muscle cells and fibroblasts, with a few studies showing their differentiation into cardiomyocytes and endothelial cells as well (40–43). Collectively, it is the progenitor cell migration from the pro-epicardial layer that dictates and coordinates cardiomyocyte proliferation and organization, electro-conduction, coronary vasculature assembly, and structural valve and chamber development (44).

While the epicardium plays an active role in the development of the embryonic heart, it exists as a dormant cell layer in the adult uninjured heart (44). However, studies have demonstrated that the genetic programme that drives epicardial-derived cell migration during development is rapidly reactivated in the adult heart in response to ischemic injury (45). Interestingly, the reactivation of the epicardium appears to occur globally throughout the heart and is not localized exclusive to the site of the injury. It was hypothesized that epicardial activation can occur due to external factors present in the pericardial fluid following myocardial infarction (46, 47).

The ability of the epicardium to orchestrate cardiac regeneration versus cardiac repair remains a highly debated topic. Studies in zebrafish and fetal non-vertebrates have identified the epicardium as source of key paracrine factors that are capable of restoring lost cardiac muscle and rescuing heart function after injury (38, 48). Conversely, epicardial activation in adult non-vertebrates following ischemic injury is limited by the number of activated progenitor cells that then differentiate exclusively to non-myocyte cells of the heart (21, 45). The mechanisms that limit regeneration despite preservation of the same embryonic gene programme are not well understood. However, if the epicardium is reactivated through an extra-cardiac paracrine milieu, perhaps modifying the paracrine microenvironment can dictate a more regenerative pathway.

Acellular scaffolds rich in cytokines and growth factors may hold the key to epicardial-driven cardiac regeneration. Using a surgically implanted epicardial patch enriched with human follistatin-like1 protein in preclinical animal models of ischemic injury, Wei and colleagues were able to document evidence of significant cardiogenesis, vasculogenesis and functional recovery in the post-MI hearts (20). Similarly, Wang et al. used a mesenchymal stem cell-loaded epicardial patch implanted one week post-MI and showed preliminary evidence of epicardial-derived progenitor cell activation and differentiation into smooth muscle cells, endothelial cells and cardiomyocytes. Here, the synthetic patch preserved MSC survival and enhanced their of expression of key cardioprotective proteins that activated the epicardium toward regeneration (21).

In addition to simple-ECM materials, more complex ECM materials derived from decellularized tissues may be leveraged towards enhanced epicardial activation. As previously discussed, the ECM serves as a natural reservoir of various growth factors and matricellular proteins that can promote tissue regeneration processes (23, 29, 49). In a preclinical porcine model of ischemia-reperfusion injury, our group has shown that the surgical implantation of an intestinal ECM scaffold on the epicardial surface of ischemic tissue resulted in increased epicardial activation (29). These findings were confirmed in a separate study where ECM scaffold therapy resulted in enhanced beta-catenin nuclear localization in the infarct area indicative of epicardial progenitor cell mobilization (34). Interestingly, both models showed evidence of enhanced vascularity in the infarct region. Since the epicardium is a known source vascular smooth muscle cells and vasculogenic paracrine factors, it is conceivable that epicardial activation following bioactive scaffold implantation can result in new blood vessel formation.

The epicardium serves as an important and necessary structure for endogenous tissue regeneration processes. Most contemporary tissue engineering strategies deliver via an intramyocardial approach and may be incapable of epicardial activation. Conversely, surgically implanted acellular scaffolds can target the epicardium directly and have been shown to enhance cardiac repair and regeneration by way of bioactive constituents that bolster epicardial activation.

### Targeting Cardiac Fibroblasts as Mediators of the Cardiac Microenvironment

Cardiac fibroblasts represent approximately 20% of the non-myocyte cell population in the heart and are directly involved in maintaining cardiac structure and remodeling (50, 51). Cardiac fibroblasts regulate the extracellular matrix microenvironment, which in turn influences surrounding cell behavior and tissue processes (52). Under normal physiological conditions, the cardiac fibroblasts are responsible for regulating ECM biology by maintaining a highly coordinated rate of turn over via specialized matrix degrading enzymes and their endogenous inhibitors (53). Due to their close association with the ECM, cardiac fibroblasts are often regarded as sentinel cells that respond to environmental stimuli and modify their behavior accordingly (54).

Under pathophysiological conditions following acute ischemic injury, cardiac fibroblasts have an important role in preserving the heart’s mechanical function through the deposition of scar tissue (50, 55). Following the initial inflammatory event that clears the ischemic area of necrotic myocytes, cardiac fibroblasts are chemically recruited to the site of granulation tissue formation and differentiate into a more contractile and secretory phenotype known as myofibroblasts (56, 57). Through a process known as reparative fibrosis, myofibroblasts contribute to wound healing by replacing lost cardiac tissue with a collagenous scar that is able to sustain ventricular load and prevent mechanical rupture (58). Although scar deposition is a necessary and adaptive reparative process, it is the continued activation of cardiac fibroblasts in the injured heart that yield more deleterious consequences to global cardiac structure and function. Understandably, therapies that mitigate scarring in the post-MI heart have been the subject of therapeutic interest.

Although activated fibroblasts have traditionally been considered a terminally differentiated cell type, there is an emerging body of evidence that suggests they are more plastic than previously appreciated. Indeed, Nobel prize winning work has shown mature dermal fibroblasts can be reprogrammed into pluripotent stem cells through invasive genetic manipulation (59). However, can cellular reprogramming or redirection of fibroblast behaviour be also achieved by changing the host-substrate environment, such as using bioactive ECM scaffolds? In a landmark study, Plikus et al. demonstrated that the fate of dermal myofibroblasts can be changed towards a regenerative adipocyte lineage by exposing cells to a new signaling microenvironment. Interestingly, these findings were replicated in myofibroblasts isolated from patients with keloids, which are characterized as pathologic scars formed by persistent myofibroblast activity (60).

Similar to keloids, the cardiac myofibroblasts in the injured heart remain continuously activated, resulting in infarct scar expansion, thinning and stiffening of the remote myocardium (53, 61–63). Here, sustained myofibroblast activity is the product of the physiologically distinct microenvironment of a healing wound characterized by complex chemical and mechanical stimuli (64, 65). Surgically implanted bioactive scaffolds may therefore target cardiac myofibroblasts directly by way of instructive paracrine and structural mediators, changing their phenotype to restore tissue homeostasis and regeneration.

**Figure 4:**
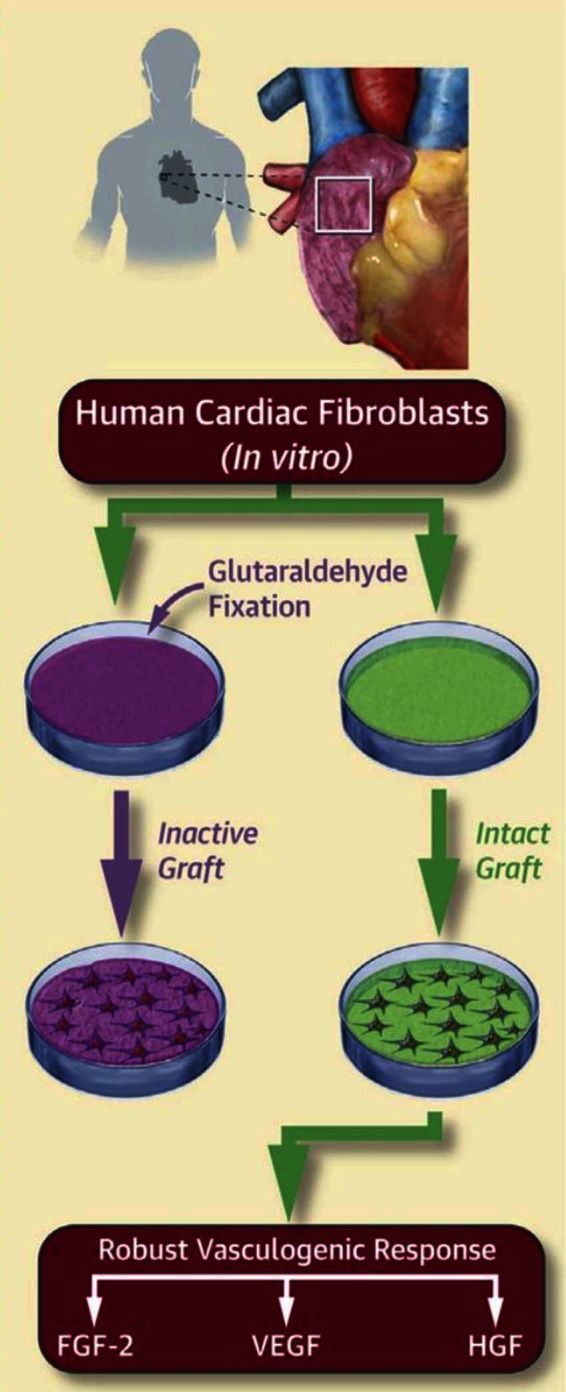
Human cardiac fibroblasts isolated from right atrial appendage were seeded onto either bioactive ECM scaffolds (intact graft) or biologically inactivated scaffolds (inactive graft). Analysis of the conditioned media revealed that cardiac fibroblasts demonstrate a robust pro-vasculogenic response specific to the bioactive ECM scaffolds (34).

Our group has recently explored this idea using cardiac myofibroblasts derived from human atria. We have shown that human cardiac myofibroblasts increase expression of key vasculogenic proteins when seeded on acellular ECM scaffolds rich with bioactive constituents (Figure 4) (34). In a rodent infarct model, we documented neovascularization with elevated concentrations of pro-vasculogenic factors in the infarcted myocardium as late as 14 weeks following ECM scaffold implantation. In a separate study, we show that ECM scaffold therapy attenuates infarct scar expansion and restores ECM homeostasis (28). Since cardiac myofibroblasts are the most abundant cell type in the infarct area (66), our collective results suggest the bioactive scaffolds may be driving the cells towards a pro-vasculogenic phenotype that create a paracrine microenvironment favoring new blood vessel formation and mitigating excessive scar deposition.

Although fibroblasts are a distinctly heterogeneous cell type, one thing that remains constant regardless of cell origin is their ability to change their behaviour and phenotype in response to different biochemical and biomechanical cues (67). Further studies characterizing the plasticity of cardiac fibroblasts to new signalling microenvironments introduced by biomaterials are warranted.

## Changing Landscape of Tissue Engineering

The future of tissue engineering will require synergy among conventional approaches that have been classically studied in a mutually exclusive manner. Combining bioactive scaffolds with other established tissue engineering strategies may hold the key to catalyzing endogenous cardiac repair mechanisms and promoting true cardiovascular tissue regeneration (68).

The strengths of bioactive scaffolds are realized not only as an effective standalone therapy, but also as a platform to deliver therapeutic agents directly to the heart. Our group has demonstrated that bioactive scaffolds can be loaded with exogenous growth factors beyond what is naturally present in the scaffolds alone (28, 33). Targeting the epicardial space may improve myocardial uptake while limiting systemic recirculation as compared to the traditional intramyocardial approach. This can mean more targeted delivery of pharmacologic therapeutics specific to cardiovascular processes.

Additionally, bioactive scaffolds may be used in conjunction with cell therapy and resolve cell engraftment and survival issues associated with intracoronary or intramuscular delivery (69). Preliminary studies have shown improved stem cell survival when tethered to ECM-based patches as well as enhanced tolerance for the hostile post-MI microenvironment (21, 70–73). The preserved biochemical and biomechanical signature of acellular bioactive scaffolds has been shown to drive cardiogenesis from seeded stem cells and augment pro-regenerative signalling (72, 74, 75). Evidence from early clinical trials support the feasibility and safety of the cell-scaffold approach (76, 77). Results from the ongoing ESCORT trial (ClinicalTrials.gov ID: NCT02057900) will provide valuable insight into the therapeutic efficacy of epicardially implanted bioactive scaffolds seeded with cardiac-committed stem cells.

Regardless of the approach, bioactive scaffolds represent a tunable platform that can be further engineered towards the specific clinical characteristics of the recipient patient. In this way, the use of acellular bioactive scaffolds complements the changing clinical landscape that is becoming increasingly focused on personalized and precise therapies.

## Conclusion

Standard therapy for ischemic heart disease patients fails to restore functional cardiac tissue. The heart contains a number of intrinsic repair processes and cell types that may be manipulated or bolstered to promote adaptive repair and regeneration. The use of acellular bioactive scaffolds for cardiac repair and regeneration is rationalized by two key points. First, bioactive scaffolds represent a unique signalling microenvironment that can target niche anatomic structures, like the epicardium, to activate endogenous repair mechanisms. Additionally, they may redirect the activity of native cardiac fibroblasts, whose fate and function is closely associated with their microenvironment, towards a more regenerative phenotype. Second, bioactive scaffolds can be leveraged as a platform for exogenous growth factors and stem cells, further maximizing their therapeutic efficacy by eliminating the common hurdles of associated with delivery. Collectively, acellular bioactive scaffolds represent a unique frontier in cardiovascular tissue engineering that may yield promising clinical outcomes.

## Author Contributions

DS, HM, and PF designed, drafted and revised the manuscript.

## Conflict of Interest Statement

The authors declare that the research was conducted in the absence of any commercial or financial relationships that could be construed as a potential conflict of interest.
